# Lab-On-Fiber Technology: A Roadmap toward Multifunctional Plug and Play Platforms

**DOI:** 10.3390/s20174705

**Published:** 2020-08-20

**Authors:** Marco Pisco, Andrea Cusano

**Affiliations:** Optoelectronic Division - Engineering Department, University of Sannio, c.so Garibaldi 107, 82100 Benevento, Italy; pisco@unisannio.it

**Keywords:** lab-on-fiber technology, optical fiber sensors, optomechanics

## Abstract

This review presents an overview of the “lab-on-fiber technology” vision and the main milestones set in the technological roadmap to achieve the ultimate objective of developing flexible, multifunctional plug and play fiber-optic platforms designed for specific applications. The main achievements, obtained with nanofabrication strategies for unconventional substrates, such as optical fibers, are discussed here. The perspectives and challenges that lie ahead are highlighted with a special focus on full spatial control at the nanoscale and high-throughput production scenarios. The rapid progress in the fabrication stage has opened new avenues toward the development of multifunctional plug and play platforms, discussed here with particular emphasis on new functionalities and unparalleled figures of merit, to demonstrate the potential of this powerful technology in many strategic application scenarios. The paper also analyses the benefits obtained from merging lab-on-fiber (LOF) technology objectives with the emerging field of optomechanics, especially at the microscale and the nanoscale. We illustrate the main advances at the fabrication level, describe the main achievements in terms of functionalities and performance, and highlight future directions and related milestones. All achievements reviewed and discussed clearly suggest that LOF technology is much more than a simple vision and could play a central role not only in scenarios related to diagnostics and monitoring but also in the Information and Communication Technology (ICT) field, where optical fibers have already yielded remarkable results.

## 1. Introduction

Lab-on-fiber (LOF) technology is a novel class of platform (the “lab”) arising from the integration of functional materials with specific mechanical, physical, and biological features onto optical fiber substrates at the microscale and nanoscale. The underlying goal is thus to provide the foundational basis for the development of multifunctional all-in-fiber devices, specifically designed and optimized for many strategic applications, ranging from optical processing and computing to environmental monitoring and life science, safety, and security tasks [1].

The extraordinary capability to combine modern nanotechnologies with nonconventional substrates, such as optical fibers, is expected to transform inert and intrinsically light-coupled platforms (optical fibers) into multifunctional plug and play optrodes, into which miniaturized labs are suitably integrated, connected, and intertwined, to provide new functionalities and achieve unprecedented performance [2,3,4,5].

Achieving these capabilities would establish the basis for a novel revolutionary class of all-fiber nanoplatforms [4,5,6], and new functionalities would create concrete opportunities for exploring new application scenarios; additionally, unparalleled performance would establish the basis for the realistic replacement of existing technologies in many strategic industrial sectors.

Optical fibers display an outstanding capability to support technological evolution and have dynamic and flexible fabrication processes. The ever-increasing demand for specialty fibers with disparate features in terms of the material composition and related properties has pushed academic and industrial researchers to develop increasingly complex processing technologies, for the creation of fibrous structures with complex functionalities [7,8,9,10].

A first milestone was undoubtedly set with the fabrication of photonic crystal fibers [11,12,13], and a relevant breakthrough in processing was achieved, as compared to the conventional steps used to create optical fibers.

Since then, many research efforts focused on creating optical fibers with new optical properties and assessing the physical mechanisms that play fundamental roles in processing. Solid-core polycrystalline semiconductor fibers were recently investigated [14], and they could potentially be used to exploit a variety of materials and achieve new and unanticipated capabilities in optics, heat, and chemical sensing. Novel imaging systems and electronic functions, such as capacitors and touch sensing, respectively, were achieved with recent developments in conductive polymer fibers [8,9].

Moreover, the unique features provided by the holed structure of photonic crystal fibers opened new avenues for achieving new functionalities and taking advantage of the ability to manage fluid and light in the same platform, by exploiting their interactions in a large number of applications [15].

In addition, studies assessed light–matter interaction mechanisms at the subwavelength scale and revealed new powerful ways to manipulate photons by enabling their slowing, trapping, localization, filtering, confinement, processing, and enhancement. New opportunities for the development of platforms with untapped functionalities could be well supported by the suitable exploitation of nanoplasmonics [16], metasurfaces [17], optomechanics [18], photonic crystals [19], ultrahigh resolution optical imaging [20], optofluidics [21], magnetoplasmonics [22], and quantum optics [23], among other things.

At this stage, the suitable combination of the unique characteristics of on-chip nanophotonics and the unparalleled features of optical fibers could realistically provide an avenue for multifunctional plug and play all-fiber platforms, designed for specific applications. This emerging field of research is called the LOF technology [1,2,3,4]. The final goal is the development of “intelligent fiber nanodevices” featuring new theranostic functionalities for precision medicine and new powerful tools for optical information processing using optical fibers.

Although nanotechnology and nanophotonics constitute the most suitable “key enabling technologies” to realistically meet the above goals, considerable research effort is needed to address important issues and overcome relevant technological barriers and limitations to translate LOF visions into reality [24,25,26,27]. Notably, material integration onto unconventional substrates with full spatial control at the nanoscale is needed, and light–matter interaction mechanisms must be identified.

## 2. Lab-On-Fiber Concept: A Technological Roadmap

### 2.1. Fabrication Evolution

**Figure 1:**
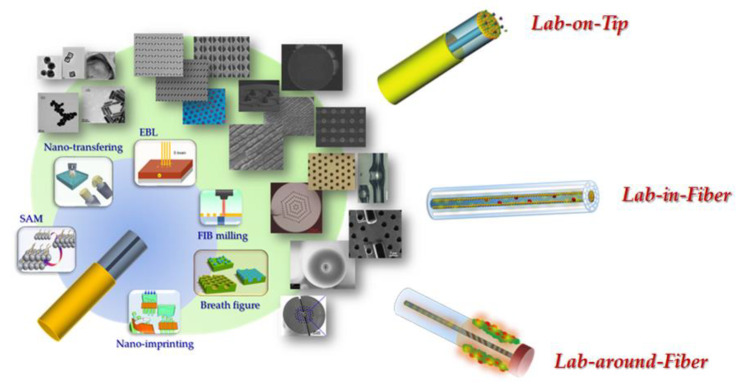
The lab-on-fiber paradigm. Reproduced with permission from Reference [5]. Copyright Wiley-VCH Verlag GmbH & Co., KGaA.

Three main LOF subclasses can be established, based on the spatial location at which functional materials are integrated, as schematically shown in Figure 1:

- **“Lab around fiber”** includes fiber platforms where functional materials are integrated onto the cylindrical surface of optical fibers;
- **“Lab on tip”** includes fiber platforms where functional materials are integrated onto the ends of optical fibers; and
- **“Lab in fiber”** includes fiber platforms where functional materials are infiltrated within the structure to create microstructured optical fibers.

Although the requirements of LOFs can be considered stringent, optical fiber technology seems prepared for a second revolution. The combination of optical fibers with materials selected for specific applications has a long tradition [28].

However, the poor spatial control and order-posed limitations associated with repeatability at the fabrication level inhibited the LOF methods, and the expected performance of LOF devices must be competitive with that of more mature technologies.

The scientific community confirmed that the assessment of suitable processing strategies is a fundamental step required to extend the paradigm of “lab-on-chip” nanophotonics to optical fibers, for the development of new plug and play fiber nanodevices.

Traditionally, micro- and nanotechnologies were developed to suitably integrate with large and planar substrates, but when dealing with optical fibers, the scenario is significantly different, especially considering their peculiarities, such as ultrasmall cross-sections, ultrahigh aspect ratios, and curved surfaces, among others.

Researchers have started to optimize nanolithography processes to ensure a high degree of spatial control, by considering materials and dimensional factors [3,4,5,6]. Many nanofabrication strategies (electrobeam lithography (EBL), focused ion beams (FIBs), interference lithography, femtosecond (FS) laser writing, nanoimprinting lithography, two-photon lithography, and tridimensional (3D) microprinting) were modified and adapted to correctly work in conjunction with optical fiber substrates, and assessments of fabrication methodologies for rapid prototyping and proof of principle demonstrations were performed [6]. EBL- and FIB-assisted fabrication, if correctly implemented for optical fiber substrates, ensures unprecedented spatial control at the nanoscale and enables the fast and reproducible development of LOF platforms [29,30]. However, limitations regarding mass production still exist and pose considerable barriers for exploitation in real scenarios. Nanoimprinting lithography, on the contrary, ensures similar performance in the control of imprinted nanostructures, with upscaling toward high-throughput configurations. FS writing, 3D microprinting, and interference lithography, typically feature cost-effective fabrication solutions combined with minor spatial control. Finally, multi photon lithography is a very intriguing technique that enables 3D nanostructuring on optical fiber substrates, which is difficult to achieve with traditional EBL and FIB approaches [3,4].

An alternative approach relies on the preliminary fabrication of nanophotonic architectures on planar substrates, followed by a second step devoted to integration into optical fibers; successful examples include soft lithography, nanoskyving, and nanomanipulation [4]. Although fabrication in a planar configuration ensures excellent quality for the nanophotonic platform, the transferring step plays a fundamental role in determining the performance of the final fiber platform [4]. These techniques are typically characterized by excellent spatial control at the nanoscale and have the capability to be scaled in mass production settings. However, additional efforts are still required to improve reproducibility and stability in transferring planar nanostructures on optical fiber substrates. Transferring failures significantly affect final performance in terms of the light coupling efficiency and overall quality of the resulting resonant platform, thus, severely limiting their exploitation in relevant application contexts.

To finally translate LOF platforms from lab curiosities into plug and play devices and components ready to be used in strategic industrial scenarios, sustainable and cost-effective mass production technologies are of fundamental importance. A valid solution could be provided by the latest advances in self-assembly routes and their extension to optical fiber substrates, as recently reviewed in [24].

The self-assembly process relies on the “autonomous organization of materials and structures” [24] and thus is inherently economical and suitable for simultaneous and appropriate operations with multiple fibers. Therefore, this approach provides a potential solution for high-throughput fabrication processes.

**Figure 2:**
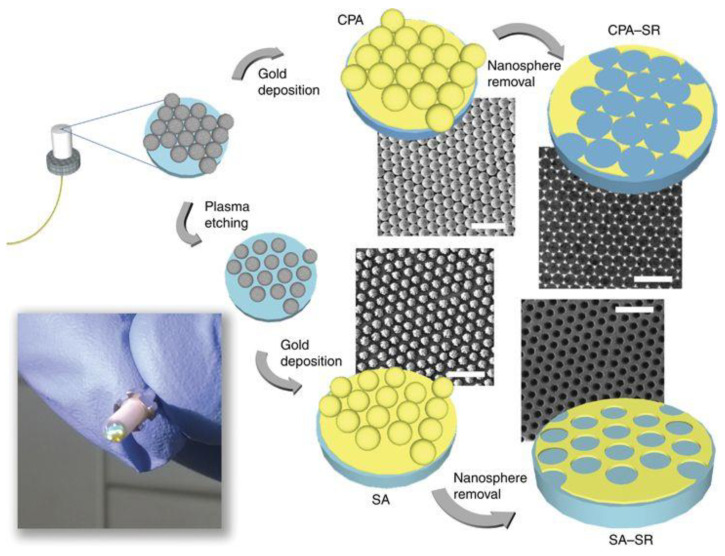
Schematic illustrating the nanosphere lithography of the fiber tip and SEM images (scale bar = 3 μm) for real samples obtained using 1-μm nanospheres corresponding to different geometric features—closely packed arrays (CPA), sparse arrays (SA), and CPA and SA sphere removal (SR). A digital picture of a fiber tip showing an iridescent nanopattern is also shown. This illustration is reproduced with permission from [33]. The article is licensed under a Creative Commons Attribution 4.0 International License: http://creativecommons.org/licenses/by/4.0/.

A valuable example was recently provided by Pisco et al., with the first demonstration of repeatable and stable plug and play fiber-optic optrodes, based on surface-enhanced Raman spectroscopy (SERS) and nanosphere lithography (see Figure 2) [31,32,33].

Self-assembly is, thus, a promising strategy for LOF platforms, but is currently limited to lab prototypes; important advancements in terms of standardization, spatial control, and long-term stability are keys for the realization of fully functional platforms that can be exploited in practical scenarios.

**Figure 3:**
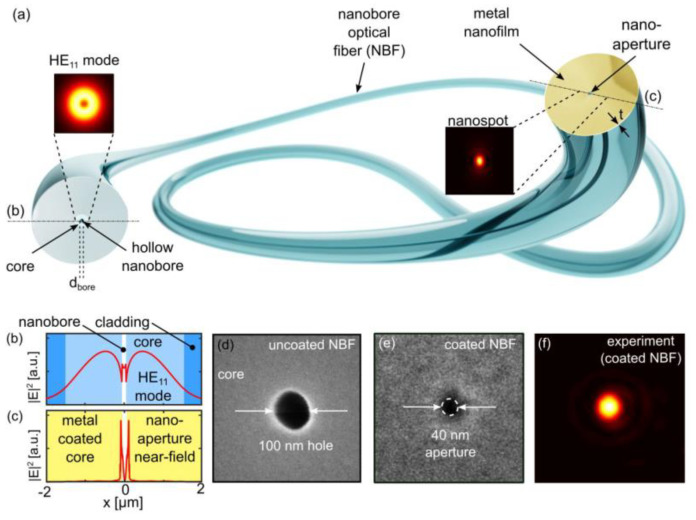
(**a**) Schematic of the concept of lithography-free nanoaperture implementation via metallic nanofilm deposition on the end face of nanobore optical fibers. (**b**) Spatial distribution of the intensity of the fundamental fiber mode along a transverse line of the empty nanobore fiber cross-section. (**c**) Distribution of the near field when the nanobore fiber is coated by a thick metal film. (**d**) Uncoated nanobore fiber featuring a hole diameter of 100 nm. (**e**) The smallest nanoaperture implemented to date (aperture diameter 40 nm; using the fiber shown in (**d**); metal: aluminum). (**f**) Corresponding measured far-field distribution showing a diffraction-limited nanospot (window size: 5 μm × 5 μm; color scales, linearly with intensity; wavelength: 633 nm). Reprinted with permission from [34]. Copyright (2019) American Chemical Society.

With a view toward mass-production processes, Tuniz et al. recently proposed the implementation of lithography-free nanoapertures onto optical fiber tips featuring novel strategies, for the development of future fiber-based nanoprobes with high spatial resolutions (see Figure 3) [34]. Due to its simplicity, scalability, and potential for large-scale production, this experimental study provides a valuable benchmark for future lab-on-a-fiber platforms by taking advantage of unconventional core/cladding geometries.

**Figure 4:**
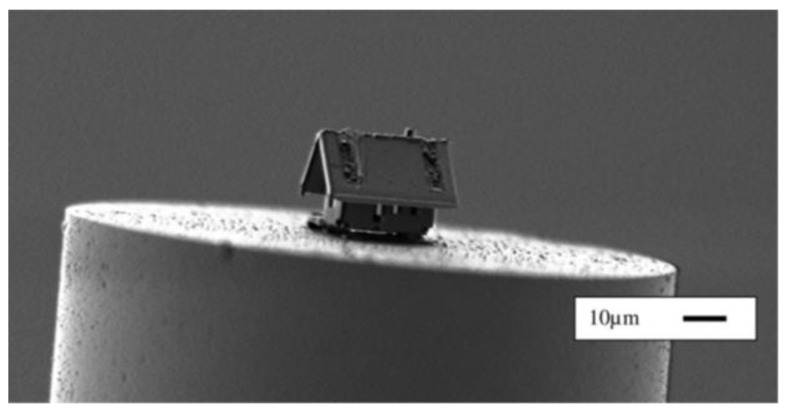
Microhouse assembled with origami and welded on top of the facet of an optical fiber. Reprinted with permission from [35]. Copyright 2019, American Vacuum Society.

A notable breakthrough in LOF fabrication was achieved with the development of new robotic fabs. Robotic arms and micro/nanotechnologies were combined to provide unique capabilities for cutting, etching, folding, assembling, and welding thin membranes on fiber facets. The enormous potential of this completely new way to conceive LOF platforms with unparalleled spatial control was recently demonstrated by Rauch et al. They demonstrated the surprising capability of their new “µ-Robotex nanofactory” approach by creating the “smallest microhouse” in the world; this microhouse was assembled on the facet of an optical fiber based on origami and welding, as shown in Figure 4 [35].

Experimental results clearly suggest that the introduction of a microrobot inside the scanning electron microscopy (SEM) vacuum chamber can provide a way to increase the scope of clean room facilities, and to build complex and smart 3D microsystems with heterogeneous materials on complex and unconventional substrates, as in the case of optical fibers. Although this approach is very promising and has the capability to significantly enhance the capability to construct real 3D labs on optical fiber substrates, notable barriers related to cost-effective and sustainable mass production settings still exist.

Despite the remaining shortcomings in fabricating reliable, reproducible, and stable LOF platforms, LOF technology reached a full maturity level in only a decade, by taking advantage of research efforts worldwide, to identify reliable fabrication techniques for the realization of complex LOF platforms involving the integration of 1, 2, and 3D nanostructures onto optical fiber substrates. Fabrication procedures involving different functional materials and very complex morphological architectures were successfully demonstrated.

### 2.2. First LOF Prototypes: New Functionalities and Unprecedented Performance

After the identification and assessment of the initial fabrication routes, research attention focused on devices with new functionalities and unparalleled performance. Successful integrations onto the optical fibers of plasmonic nanostructures [36,37], photonic crystals [38], ring resonators [39], optomechanical microcavities [40], metallodielectric nanoarchitectures [41,42,43], and dielectric block surface-wave resonators [44] were recently reported. These first proofs of concept provide the basis for the development of multifunctional LOF platforms, such as plasmonic label-free optical fiber nanosensors [43,45,46], SERS nanoprobes [33,47], advanced photonic nanoresonators [39,44], ultrahigh-sensitivity acoustic transducers [48], optical fiber tweezers [49,50], and optical fiber metatips [51].

Most previous studies were motivated by the needs of advanced diagnostic platforms for life science applications [3,52,53,54,55,56]. In this context, LOF optrodes represent a major technological breakthrough, with an enormous impact on science, industry, and society; the devices can be used in in vivo biomolecular recognition at pM levels, and provide new diagnostic features.

**Figure 5:**
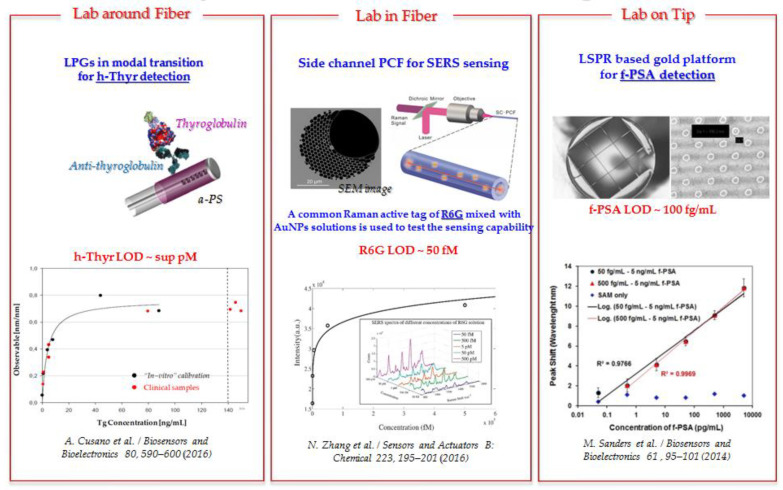
Representative results for LOF bioprobes with detection limits approaching the pM and fM regimes. Adapted with permission from [57] and [45]. Copyright © Elsevier B.V.

This vision is well supported by LOF platforms, due to the advantages related to their ease of integration with medical catheters and hypodermic needles, compared to the use of lab-on-chip platforms. In this context, many research projects developed LOF biosensors, with limits of detection approaching the pM and fM levels; these studies provided the basis for a novel generation of diagnostic in vivo liquid and tissue biopsies [5]. Representative results are reported here and schematically shown in Figure 5:

- SERS lab-in-fiber platforms with enhancement factors up to 10^7^ were recently demonstrated for detection at fM levels [57].
- Label-free lab-on-tip optrodes with gold nanopillars integrated on the fiber tips and local plasmonic resonance were shown to achieve a surprisingly good limit of detection (fM) in a clinically relevant scenario involving free prostate-specific antigen (f-PSA) as a biomarker for prostate cancer [45].
- Lab-around-fiber bioprobes specially designed for thyroglobulin assays (biomarkers for thyroid cancer) demonstrated pM detection levels, thus, creating the technological basis for the application of LOF platforms in conjunction with fine-needle aspiration biopsy platforms, to assess the metastatic nature of locoregional lymph nodes [5].

Further sensitivity improvements were recently achieved in the case of label-free “lab-on-tip” nanobioprobes, for controlled integration on the nanostructured tip of single-mode optical fibers in multiresponsive and functionalized microgels [58].

Moreover, in life science scenarios, new functionalities were introduced by taking advantage of the ingenious design of nanostructured fiber facet tips, to manipulate the light emitted from fiber termination. In this context, by using suitable fabrication methodologies optimized for optical fibers, LOF tweezers were demonstrated in different configurations, as recently reviewed [55]. Spherical and conical architectures were verified using very simple and cost-effective fabrication strategies (polishing [59], chemical etching [60], and thermal pulling [61]), and more complex designs require techniques with high spatial resolutions, such as focused ion beam and two-photon lithography, which are generally more expensive and time consuming than the traditional methods [49,50]. Finally, Ribeiro et al. proposed a guided-wave-assisted photopolymerization process for the cost-effective and rapid fabrication of plug and play fiber-optic tweezers [54,62].

The main takeaway of previous studies was that needles that employ multifunctional LOF platforms would likely be achieved in the near future. These “intelligent needles” could provide real added value information about clinically relevant parameters for the human body, diseases, and progression/regression behavior for given therapies, thus, providing a new approach in precision medicine. Similarly, it is not unreasonable to envision LOF-assisted smart catheters that are able to identify cancer cells and quantify cancer biomarkers in close proximity to a tumor, thus, establishing the basis for new theranostic platforms that can be used in in vivo applications.

**Figure 6:**
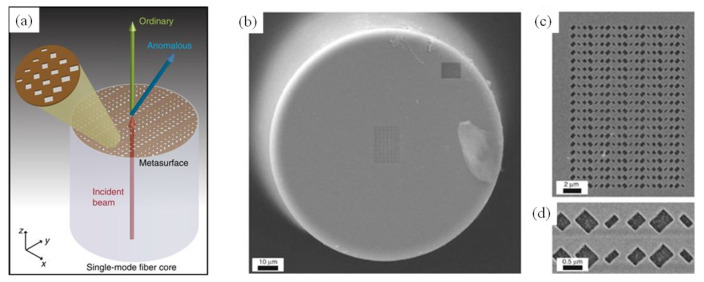
(**a**) Schematic representation of an optical fiber metatip. A plasmonic metasurface is fabricated on the optical fiber end facet. The metasurface displays a linear-phase profile at the wavefront of a given component of an impinging beam. As a result, the transmitted beam is split into an ordinary (copolarized) component propagating in the incidence direction and an anomalous component (with generally different polarization) undergoing a phase-gradient-induced steering of an angle. (**b**) SEM image of a fabricated sample with the entire fiber cross-section shown. (**c**,**d**) Two magnified details showing the entire metasurface and two unit cells. Reproduced with permission from [51]. The article is licensed under a Creative Commons Attribution 4.0 International License: http://creativecommons.org/licenses/by/4.0/.

Considering new ways to manipulate light using optical fibers, an important milestone that was recently set on the basis of LOF roadmap, relied on the first integration of a phase-gradient plasmonic metasurface on the facet of a single-mode optical fiber (see Figure 6) [51]. This proof of principle is expected to significantly accelerate the applicability of optical metasurfaces in real-world scenarios, thus, creating new market opportunities. Additionally, such an approach would provide the foundational basis to significantly broaden the functionalities and application perspectives of plug and play LOF platforms.

Potential direct applications could include use in active beam profilers, spatial light modulators, and fiber-optic tweezers. The capability to translate the metasurface-assisted ‘flat-optics’ concept onto optical fibers would play a fundamental role in biomedical imaging, scanning near-field optical microscopy, and single-molecule detection. Nevertheless, the exploration of metasurface-assisted LOF platforms with properly conceived activity mechanisms (e.g., electro-optic, magneto-optic, and fluid-based mechanisms) could aid in the development of active optical switches and active fiber-optic metadevices.

**Figure 7:**
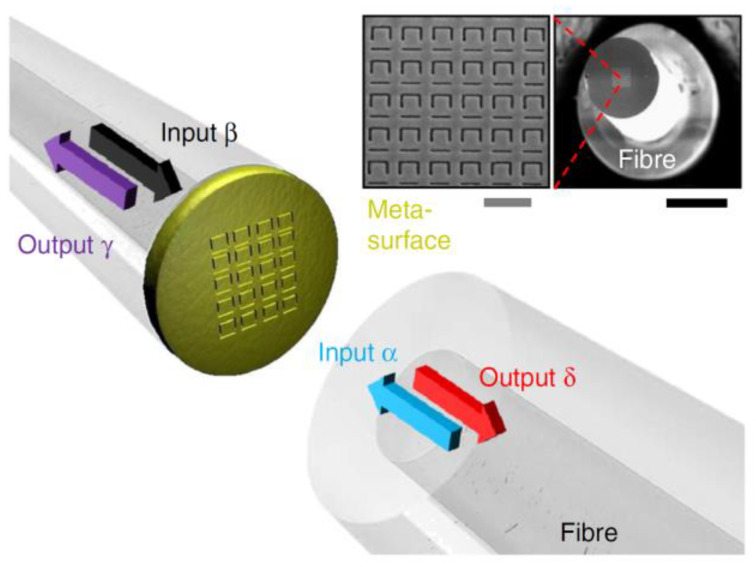
Coherent interaction of light with a metasurface. Coherent optical input signals α and β interact on a metasurface absorber and generate output signals γ and δ. The metasurface was fabricated by nanostructuring the central 25 × 25 μm^2^ of a 70-nm-thick gold layer covering the cleaved end face of a polarization-maintaining single-mode silica fiber (inset scanning electron microscope images, black scale bar of 100 μm, gray scale bar of 1 μm). Reproduced with permission from [63]. The article is licensed under a Creative Commons Attribution 4.0 International License http://creativecommons.org/licenses/by/4.0/.

A recent example of the above concept was provided by Zeludev et al., who developed an LOF-assisted metadevice for optical signal processing; the device is fully compatible with telecommunication optical fibers and the related components (see Figure 7) [63].

Coherent transparency and coherent absorption were proposed here to achieve nonlinear signal processing, and these methods included XOR, AND, and NOT operations. The corresponding proof of principle strongly reinforced the concept that ready-to-use plug and play LOF metadevices would provide effective solutions for quantum information networks.

Fiber-optic metatips could also have an enormous impact on imaging, especially, in life science applications, through the reliable integration of metalenses onto optical fiber substrates, thus, leading to next-generation fiber-optic imaging capabilities, with unparalleled spatial resolutions and miniaturization levels.

**Figure 8:**
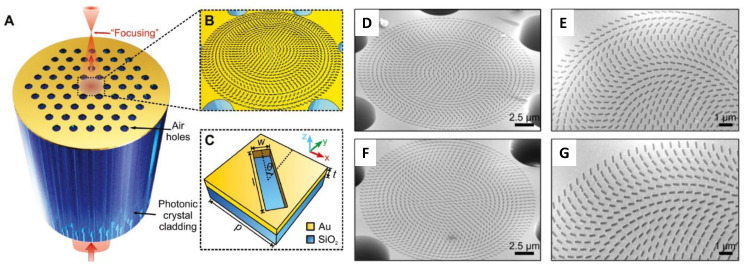
(**A**,**B**) Schematics of in-fiber metalens on photonic crystal fiber (PCF) and (**C**) unit element of PCF metalens. SEM images of fabricated PCF metalens for numerical apertures of (**D**,**E**) 0.37 and (**F**,**G**) 0.23. Reproduced with permission from [64]. The article is licensed under a Creative Commons Attribution 4.0 International License: http://creativecommons.org/licenses/by/4.0/.

Yang et al. reported an ultrathin optical metalens directly patterned on the facet of a photonic crystal optical fiber (see Figure 8) that could achieve light focusing in the telecommunication regime [64]. In-fiber metalenses with focal lengths of 28 μm and 40 μm at a wavelength of 1550 nm were demonstrated with maximum enhanced optical intensities as large as 234%.

The integration of metalenses and optical fibers could considerably improve the acquisition of high-resolution images of internal organs in relevant clinical applications, especially considering the difficulties associated with optical aberrations and the trade-off between the transverse resolution and depth of focus, which significantly limits many applications.

Metalenses coupled with optical coherence tomography (OCT) tools are expected to provide the key components for the next generation of smart nanoendoscopes for in vivo imaging, leading to spatial resolutions that are not possible with conventional platforms.

**Figure 9:**
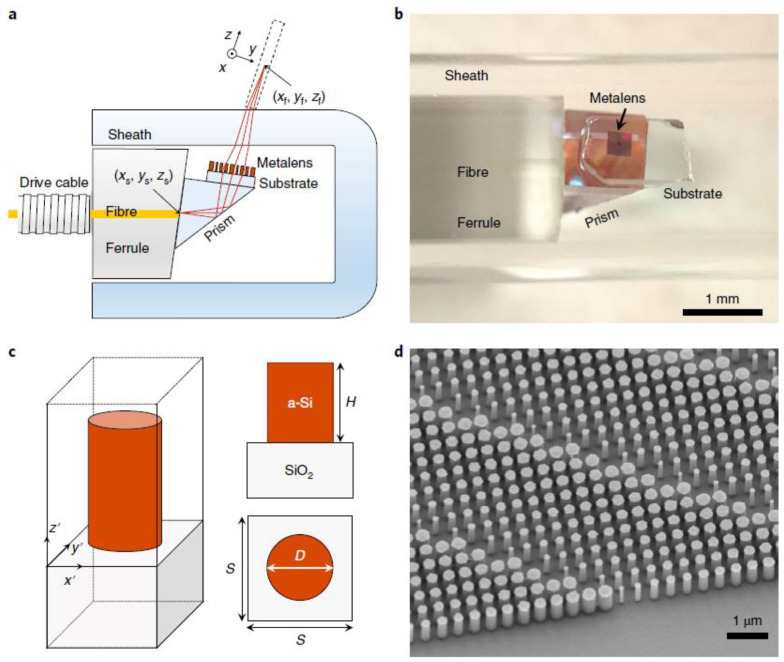
Nano-optic endoscope. (**a**) Schematic of the nano-optic endoscope. (**b**) Photographic image of the distal end of the nano-optic endoscope. (**c**) Schematic of an individual metalens building block consisting of an amorphous silicon (a-Si) nanopillar on a glass substrate. (**d**) Scanning electron micrograph image of a portion of a fabricated metalens. Reproduced with permission from [65]. Copyright © 2018, Springer Nature.

**Figure 10:**
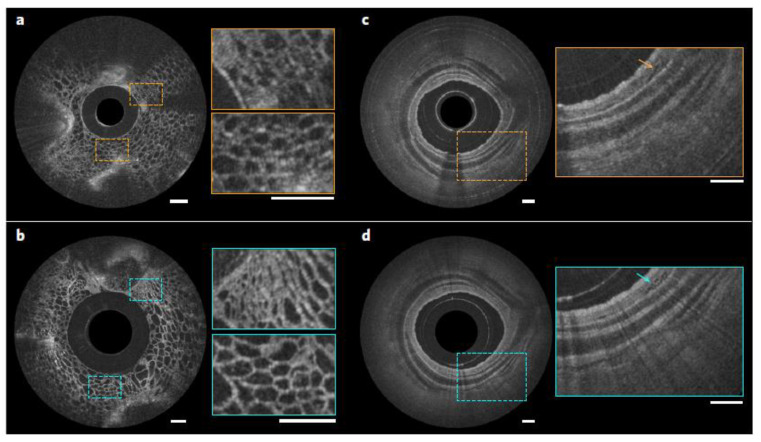
Comparison of the OCT images acquired using a nano-optic endoscope and a conventional OCT catheter. (**a**,**b**) OCT images of fruit flesh (grape) obtained using a ball lens catheter (**a**) and nano-optic endoscope (**b**). (**c**,**d**) Ex vivo images of a swine airway using a ball lens catheter (**c**) and nano-optic endoscope (**d**). The arrows indicate fine glands in the bronchial mucosa. All scale bars are 500 μm. Reproduced with permission from [65]. Copyright © 2018, Springer Nature.

A proof of principle of a metalens-assisted nanoendoscope was recently provided by Capasso et al., through the integration of a metalens. This device has the ability to modify the phase of incident light at the subwavelength level and can be used in endoscopic OCT catheters (called a nano-optic endoscope; see Figure 9) to achieve near-diffraction-limited imaging by negating nonchromatic aberrations [65]. The endoscopic imaging of resected human lung specimens and sheep airways was performed in vivo, and superior performance was achieved, as compared to that of commercial OCT endoscopes (see Figure 10).

The versatility and design flexibility of nano-optic endoscopes make them ideal for new uses, which is not possible with conventional catheter fabrication techniques. The ability of metalens-assisted OCT tools to obtain high-resolution images of subsurface tissue structures in vivo is likely to increase the clinical utility of OCT in the detection, diagnosis, and monitoring of diseases.

Overall, LOF technology provides a solid basis for the generation of future technologies that incorporate multifunctional plug and play LOF nanodevices for broad use in society and industry.

## 3. Merging LOF with Optomechanics

Most of the platforms demonstrated to date under the LOF umbrella rely on plasmonic interactions that occur when metallic nanostructures are integrated onto optical fibers [4]. In the past decade, with the optimization of fabrication processes for the development of 3D nanostructures that can be suitably integrated onto optical fiber substrates, we saw an increased interest in merging LOF technology and optomechanics [18,66,67], to enhance the application range of LOF platforms.

The envisioned synergy would enable the engineering and integration of optomechanical nanosystems with optical fiber technology, for the manipulation of light flow through powerful optomechanical interactions, and the detection of weak mechanical effects (small forces, displacements, masses, accelerations, and acoustic and ultrasonic waves); this approach achieves unprecedented performance when compared to that of conventional technologies [67].

The first demonstrations of miniaturized optomechanical LOF devices date back to 2007 when Kilic et al. [68] proposed a micromachined optical fiber microphone consisting of a tiny Fabry–Pérot microcavity formed by a photonic crystal diaphragm placed a short distance from a mirror deposited at the tip of a single-mode optical fiber. This miniaturized fiber microphone was used to measure air pressures with a limit of detection as low as 18 µPa/√Hz at 30 kHz. Then, the same authors fabricated an optical hydrophone based on a compliant membrane suitably integrated onto an optical fiber tip, to be exploited in underwater applications, for measuring acoustic pressures with a resolution close to that of background noise in the ocean over a bandwidth of 10 kHz [69].

In 2006, Iannuzzi et al. first launched the so-called “fiber-top cantilever” for multifunctional optomechanical platforms, through the integration of a cantilever on the tip of single-mode optical fibers [70,71]. The proposed approach relied on the inherent Fabry–Pérot cavity formed between the fiber and the cantilever as the main transduction mechanism. Applications of fiber-top cantilever probes were demonstrated for sensing and atomic force microscopy applications [72]. First, fiber-top probes were initially fabricated through focused ion beam milling, but Iannuzzi et al. proposed a more versatile and sustainable fabrication process involving laser micromachining for large optical substrates, such as glass ferrules [73,74]. This successful translation transformed the initial “fiber-top cantilever” technology into a “ferrule-top technology”. Additionally, a top-down approach based on align-and-shine photolithography [75] was proposed to fabricate low-mass, gold, fiber-top cantilevers.

Following these technological improvements, a variety of novel fiber-optic microsensors were demonstrated, including pressure sensors [76], acoustic emission detectors [77], and humidity and force transducers [78,79]. Sensing systems based on micromechanical transducers directly created on the top of a glass ferrule were also demonstrated for surface topography reconstruction and photoacoustic spectrometry [80].

By observing the results of these pioneering studies, the enormous potential associated with combining LOF and optomechanical technologies, for the development of a novel class of fiber devices with new functionalities and unparalleled performance, could be observed.

**Figure 11:**
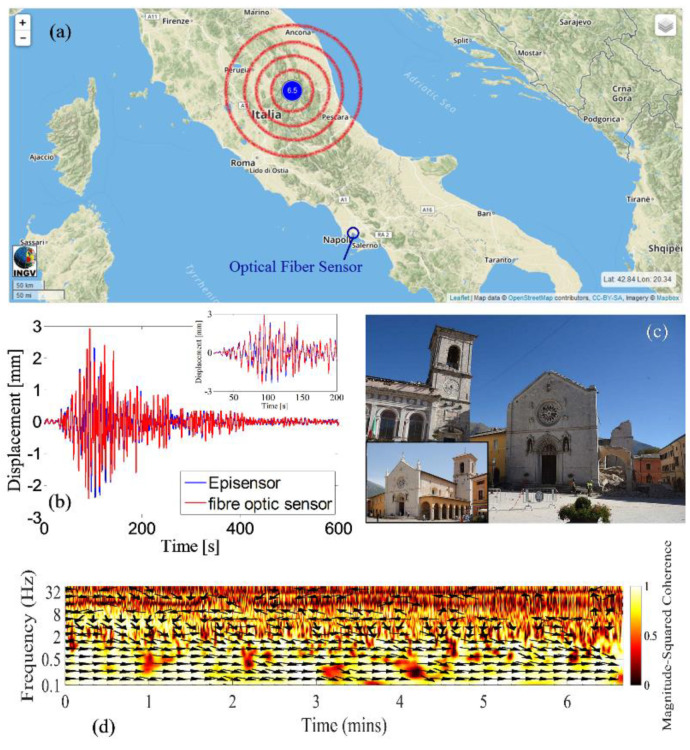
(**a**) Map of central Italy highlighting the Norcia earthquake epicenter and seismic station location (the map from http://cnt.rm.ingv.it/ was released under a CC-BY-SA license https://creativecommons.org/licensesbysa/3.0/). (**b**) The ground displacement traces represent the recordings of the LOF sensors and the reference sensor during the Norcia earthquake. (**c**) Photograph of St. Benedict Cathedral in Norcia after the disruptive Norcia earthquake in 2016; in the inset, a picture of the Cathedral before the earthquake is shown (the picture is released under a CC-BY-SA license https://creativecommons. org/licenses-sa/3.0/by Wikimedia). (**d**) Wavelet coherence between the optical and episensor displacement signals over a 400-s window. Reproduced with permission from [81]. The article is licensed under a Creative Commons Attribution 4.0 International License: http://creativecommons.org/licenses/by/4.0/.

As an example, a ferrule-top approach was recently used to integrate a complex micromechanical structure featuring a dual-beam cantilever with a properly designed mass onto an optical fiber tip. The innovative optomechanical structure was designed to act as a seismic accelerometer with competitive performance in seismic applications, when compared to that of commercial platforms [81]. Prototypes were fabricated, and their responsivity was preliminarily measured in the laboratory at resolutions down to 0.44 μg/√Hz, over a 3-dB frequency band of 60 Hz. Successively, LOF seismic accelerometers were integrated into a conventional seismic station and used for seismic surveillance applications. During field trials, LOF sensors registered the ground acceleration associated with the seismic sequence that occurred in 2016 in central Italy (see Figure 11). The wave traces were compared with the recordings of a traditional sensor used as a reference, and the excellent performance of the LOF sensors in seismic wave detection was demonstrated. In addition, the characteristics of LOF sensors, in terms of their light weight, small size, and electromagnetic interference immunity, allow for simple installations when compared to those for traditional sensors. The overall performance and effectiveness in detecting ground acceleration during earthquakes, combined with the benefits of optical fiber technology, make the proposed seismic accelerometers a promising alternative to conventional seismic accelerometers.

Recently, the same group demonstrated that micromechanical structures on the optical fiber tip, in the shape of cantilevers or membranes, could be used to implement fiber-tip accelerometers at high frequencies (in the kHz range) by carefully managing the intrinsic trade-off between sensitivity and bandwidth [82,83].

A significant milestone in optomechanical-assisted LOF platforms was achieved by the first demonstration of a ferrule-top “nanoindenter” [84]; such devices are now commercially distributed. The indenter uses a force probe composed of a cantilever suspended on the top of an optical fiber hosted by a glass ferrule. A Fabry–Pérot interferometric cavity is created between the fiber end and the cantilever free end. A small tip on the external surface of the cantilever comes in contact with the sample being tested. By imposing a small oscillatory load on the probe and measuring the cavity length via an interferometric technique, the optomechanical instrument allows for the extraction of the indentation depth at the frequency of oscillation and thus the local elastic and viscous moduli of the sample being tested. The resulting indenter instrument was proposed to probe the viscoelastic properties of soft hydrated tissues and biomaterials [85,86,87,88], but it was also used in completely different fields, such as cultural heritage assessment and in the laboratory reconstruction of historical oil paints [89].

**Figure 12:**
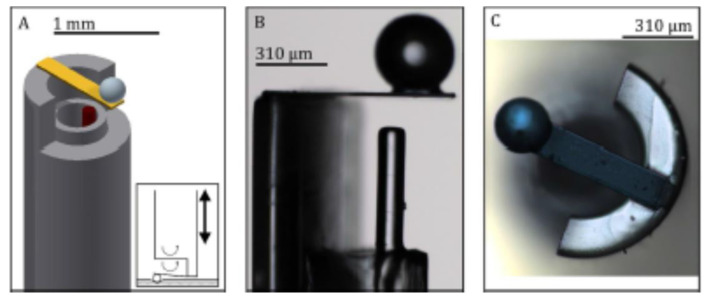
(**A**) Schematic of the force probe. The external capillary has a diameter of 1 mm and wall thickness of 0.21 mm, and the internal capillary, which hosts the optical fiber, has a diameter of 0.55 mm and a wall thickness of 0.075 mm. The inset shows a schematic diagram of the indentation procedure. (**B**) Microscope image of the probe showing the interferometric cavity. (**C**) Top view of the sensor. Reproduced with permission from [90]. The article is licensed under a Creative Commons Attribution 4.0 International License: http://creativecommons.org/licenses/by/4.0/.

A similar prototype was recently developed to operate in situ on a needle [90]. As shown in Figure 12, the cantilever was mounted on an 8-cm-long borosilicate capillary, and the optical fiber was hosted by another borosilicate capillary inserted inside the first capillary. The probe could be used to perform minimally invasive measurements, retrieve the mechanical properties of a biological tissue in situ, and under the mechanics of physiology and tissue engineering [90].

These first successful demonstrations, combined with successful industrial applications of the developed platforms, provide the basis for further developments involving complex and powerful optomechanical LOF platforms for in vivo biomedical processes.

Giaquinto et al. reported a cavity-enhanced LOF optrode consisting of a microgel film sandwiched between two gold layers on an optical fiber tip. The combination of the optical resonant effects and microgels gave the optrode the unique ability to work as both a sensor for detecting small molecules and a nano-optomechanical actuator triggered by light. Notably, the light could be used by the fiber, specifically, by exploiting thermoplasmonic heating and microgel thermoresponsivity mechanisms, to control the optical cavity features. Thus, providing a basis for the development of active nano-optomechanical actuators directly integrated into fiber tips [91].

**Figure 13:**
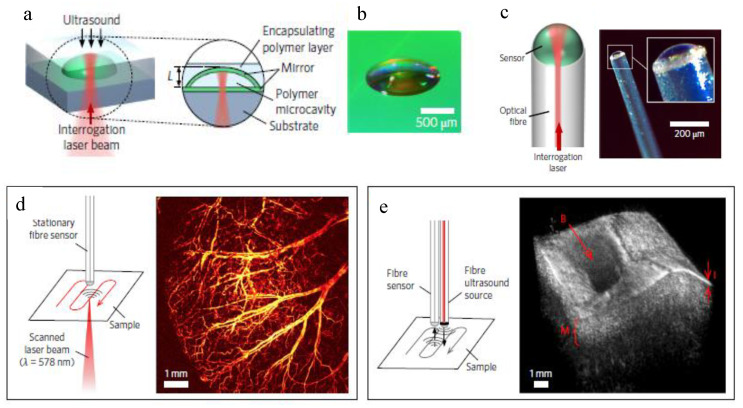
(**a**) Schematic of the plano-concave optical microresonator. (**b**) Photograph of polymer microcavity prior to the application of the encapsulating polymer layer. (**c**) Schematic diagram (left) and photograph (right) of the optical fiber microresonator sensor. (**d**) Schematic diagram of a fiber microresonator sensor-based optical-resolution photoacoustic microscopy experiment and an image of mouse ear vasculature in vivo. (**e**) Schematic diagram of the all-fiber pulse-echo ultrasound experiment and 3D pulse-echo ultrasound image of an ex vivo porcine aorta. Reproduced with permission from Macmillan Publishers Ltd. [92]. Copyright 2017.

Guggenheim et al. developed novel plano-concave polymer microresonators [92] integrated onto optical fiber tips. The sensors were based on a polymeric plano-concave microcavity. The cavity was composed of two reflective mirrors embedded in a layer of matching polymer, as illustrated in Figure 13a–c. The cavity was constructed by adding a droplet of ultraviolet (UV) curable polymer onto a dielectric mirror. The droplet stabilized to form a smooth spherical cap under surface tension and was subsequently cured under UV light. Then, a second dielectric mirror coating was applied. The additional encapsulating layer was formed by the addition and curing of a matching polymer to create an acoustically homogeneous planar structure.

This technique enabled the realization of advanced LOF ultrasound transducers with unprecedented performance (i.e., omnidirectionality; bandwidth up to 40 MHz, and noise-equivalent pressure as low as 2.1 mPa/√Hz), when compared to that of traditional piezoelectric transducers [92].

These ultrasound probes were successfully demonstrated in high-resolution photoacoustic and ultrasound imaging in clinically relevant scenarios.

In Figure 13d, we display an optical-resolution photoacoustic microscopy image of a mouse ear acquired in vivo, using an optical fiber sensor. From the image, the microvasculature at the level of individual capillaries could be observed. The image was obtained by scanning the test area with a pulsed laser beam and recording the photoacoustic signals at each scan point, with the optical fiber sensor placed at a fixed position. Specifically, the fiber sensor was located at the center of the scan area (8 mm × 8 mm), at a distance of 1.2 mm from the mouse ear skin. In Figure 13e, we display a 3D ultrasound image, acquired ex vivo, of a porcine aorta sample. The image was obtained by scanning the tissue with a fiber-based pulsed laser ultrasound source and a fiber microresonator sensor. The sensor records the returning echoes from the subsurface tissue structures and reconstructs the three-dimensional tissue image.

The above achievements suggest that in the near future, the development of smart needles with unprecedented functionality and integration and miniaturization levels and that are able to monitor clinically relevant parameters in real time, directly inside the human body, could provide a way to monitor disease appearance and progression/regression. Notably, cancer cells could be imaged and differentiated, and cancer biomarker concentrations in close proximity to a tumor could be quantified.

Another relevant milestone was achieved in 2017 when Yao et al. proposed the use of a 3D printing approach to create polymeric optomechanical structures in 3D arbitrary formats, on the end face of fiber-optic ferrules [93]. Notably, a sustainable and cost-effective fabrication root for the novel generation of optomechanical-assisted LOF platforms, designed for specific applications was created. The optical 3D μ-printing system consisted of a UV source, a digital mirror device (DMD) for optical pattern generation, projection optics for downscaling optical images and a digital camera for precise position determination. In reality, the addition of advanced polymeric structures on the fiber tip was previously achieved in different applications through multiphoton polymerization. Various refractive lenses, photonic crystals, and Fabry–Pérot interferometer sensors were also previously fabricated [94,95]. The authors, with a similar approach, fabricated several optomechanical structures, namely, suspended-mirror devices (SMDs), on fiber tips. The optical exposure process was used to reproduce 3D SMD images in successive slices, and the predefined SMDs were fabricated through the photopolymerization of the SU-8 photoresistors. The application of such fabricated ferrule-top SMDs as displacement microsensors was experimentally demonstrated [93].

In 2018, Yao et al. presented an improved version of optical µ-printing technology, which could to directly fabricate suspended microbeams on the end face of an optical fiber [96]. To improve the deposition of SU-8 on the fiber tip, they used an ultrasonic nozzle for spray coating the fiber tip. By adjusting the spray parameters, such as the gas pressure associated with the ultrasonic nozzle, the scanning velocity, the pumping rate of the syringe pump, and the distance between the nozzle and substrate, the thickness of the single-layer SU-8 film could be accurately defined.

**Figure 14:**
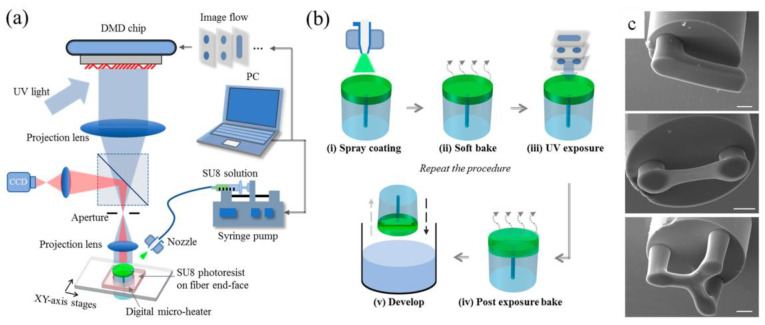
(**a**) Schematic diagram of optical 3D µ-printing technology. (**b**) Steps involved in printing optical fiber-tip sensors. (**c**) Scanning electron microscope (SEM) images of SU-8 optomechanical cavities in optical fiber tips. All scale bars are 20 μm. Reproduced with permission from [95]. The article is licensed under a Creative Commons Attribution 4.0 International License: http://creativecommons.org/licenses/by/4.0/.

As shown in Figure 14a, to fabricate a 3D microstructure, a thin layer of SU-8 was preliminarily deposited on an optical fiber by spray coating; the process is shown in Figure 14b. The film was soft baked in situ to remove solvent. Then, the sample, which was accurately positioned with the assistance of a digital camera, was subjected to UV exposure and subsequent in situ baking, by using an integrated digital microheater. These steps were automatically repeated for the fabrication of the successive layers of the 3D microstructure, and finally, the sample was developed by using propylene glycol methyl ether acetate (PGMEA).

In Figure 14c, the SEM images of three SU-8 optomechanical cavities fabricated on the end faces of optical fibers are shown. Such optical fiber-tip sensors were demonstrated to detect changes in the surrounding refractive index and the gas pressure of the ambient environment with sensitivities of 917.3 nm/RIU and 4.29 nm/MPa, respectively.

As noted by the same authors, as the Young’s modulus of SU-8 and similar photoresists is relatively low (with respect to those of glass and silicon), optical 3D printing is a valuable option for fabricating optomechanical cavities with low resonant frequencies or critical damping. The combined control of the resonant frequency and damping can indeed be used to tailor the damping response of optomechanical accelerometers [83].

By following this fabrication approach, an optical fiber-acoustic sensor based on a microscale suspended polymer microdisk was directly printed in situ on the end face of a standard single-mode optical fiber. The microdisk was suspended by microbeams developed with a spiral structure, to improve the sensitivity to acoustic waves. An acoustic sensor with a high sensitivity of 118.3 mV/Pa and low noise-equivalent acoustic signal of 0.328 µPa/Hz^1/2^ at audio frequencies, was experimentally demonstrated [97].

As the usability of the constituent materials and versatility of the design process have improved, 3D microprinting technology has become a cost-effective strategy for fabricating multifunctional plug and play devices with optomechanical cavities, and precise and controlled geometries, onto optical fiber substrates.

## 4. Conclusions

In this review, we provide an overall picture of LOF technology, by presenting the main milestones achieved along the LOF technological roadmap, for the development of flexible and multifunctional plug and play fiber-optic platforms designed for specific applications.

The first milestones in the LOF roadmap involved assessments of reliable and cost-effective nanofabrication strategies on optical fiber substrates. Several nanofabrication methodologies could be used to effectively integrate functional materials and nanoarchitectures with precise and full spatial control.

With these efforts, many new functionalities were demonstrated, thus, allowing new fields and market options to be explored. The preliminary examples provide evidence for the realistic replacement of existing technologies with engineered LOF platforms, in many strategic sectors.

Despite the nontrivial nature of optical fiber substrates, currently, it is possible to conceive arbitrary 3D objects being integrated onto optical fiber tips, as demonstrated through the construction of the smallest microhouse in the world. Creating any arbitrary object on an intrinsically light-coupled substrate, such as an optical fiber, requires the management of a large set of degrees of freedom for nanophotonic, optomechanical, and fluid systems in a single platform, thus, pushing light–matter interactions to the known limit.

Considering the technological advancements achieved so far, new achievements are soon expected and might be related to the fundamental transition from the “component and device” level to the “system” level, where advanced plug and play LOF platforms are conceived and integrated into complex systems designed for specific applications.

Clear evidence of this technological transition is provided by the first demonstrations of LOF-assisted OCT nanoendoscopes and in vivo ultrasonic imaging tools, specially designed for in vivo imaging. Similar considerations can be easily extended to the first optical systems that incorporate LOF metadevices designed for optical processing and computing.

In the future, the development of intelligent needles is expected by combining LOF platforms specialized for biomanipulation, tissue and liquid biopsies, locoregional echographic assessment, and drug delivery in precision medicine and clinically relevant scenarios.

Although most of the target applications were life science applications, considering the ease of integration of LOF platforms into catheters and hypodermic needles, there is no real limit for the exploitation of such technology in numerous other industrial scenarios.

In this context, optomechanics-assisted LOF technologies seem to be the most promising route for applications in new fields and alternative markets.

The merging of mechanical micro- and nanocavities onto optical fiber substrates will promote the development of plug and play LOF platforms that can be used in force and pressure sensing, or other related physical measurement tasks (e.g., for acoustic waves or acceleration). At this stage, this technological integration has not yet reached its full potential.

In this context, future milestones include the identification of new technological steps aimed at efficiently incorporating integrated mechanical and optical systems, to actively change and control the resulting optical signals.

LOF technology could potentially play a central role in not only the scenarios related to diagnostics and monitoring in life science applications, but also in applications in the ICT field, where optical fibers have yielded remarkable accomplishments.
